# Macrophages Improve Survival, Proliferation and Migration of Engrafted Myogenic Precursor Cells into MDX Skeletal Muscle

**DOI:** 10.1371/journal.pone.0046698

**Published:** 2012-10-02

**Authors:** Pierre-François Lesault, Marine Theret, Mélanie Magnan, Sylvain Cuvellier, Yiming Niu, Romain K. Gherardi, Jacques P. Tremblay, Luc Hittinger, Bénédicte Chazaud

**Affiliations:** 1 CRCH Universitaire de Québec, Sainte-Foy, Quebec, Canada; 2 INSERM, U955, Créteil, France; 3 Univ Paris Est-Créteil, Créteil, France; 4 INSERM, U1016, Institut Cochin, Paris, France; 5 CNRS, UMR8104, Paris, France; 6 Univ Paris Descartes, Paris, France; University of Minnesota Medical School, United States of America

## Abstract

Transplantation of muscle precursor cells is of therapeutic interest for focal skeletal muscular diseases. However, major limitations of cell transplantation are the poor survival, expansion and migration of the injected cells. The massive and early death of transplanted myoblasts is not fully understood although several mechanisms have been suggested. Various attempts have been made to improve their survival or migration. Taking into account that muscle regeneration is associated with the presence of macrophages, which are helpful in repairing the muscle by both cleansing the debris and deliver trophic cues to myoblasts in a sequential way, we attempted in the present work to improve myoblast transplantation by coinjecting macrophages. The present data showed that in the 5 days following the transplantation, macrophages efficiently improved: i) myoblast survival by limiting their massive death, ii) myoblast expansion within the tissue and iii) myoblast migration in the dystrophic muscle. This was confirmed by *in vitro* analyses showing that macrophages stimulated myoblast adhesion and migration. As a result, myoblast contribution to regenerating host myofibres was increased by macrophages one month after transplantation. Altogether, these data demonstrate that macrophages are beneficial during the early steps of myoblast transplantation into skeletal muscle, showing that coinjecting these stromal cells may be used as a helper to improve the efficiency of parenchymal cell engraftment.

## Introduction

Normal adult skeletal muscle fully regenerates due to the properties of satellite cells which are the main contributors of new myofibre formation [1]. In many muscular dystrophies, primary defect, i.e. lack of a structural protein, confers instability to the myofibres. This leads to continuous degeneration–regeneration cycles, which are associated with inflammation, fibrosis, adiposis and eventually muscle atrophy [2]. Several new therapeutic strategies are envisaged for the treatment of muscle dystrophies, among which cell therapy using myogenic precursor cells (MPCs). Intramuscular transplantation of such cells is particularly of interest for focal diseases such as Oculopharyngeal Muscular Dystrophy, Facioscapulohumeral Muscular Dystrophy or sphincter insufficiency [3], [4]. Indeed, targeting the whole vasculature requires systemic delivery of the cells and their homing to the diseased areas. Since the first assays in 1989 [5], [6], various cell types have been tested to recover dystrophin expression in dystrophinopathic muscle [7], [8]. However, whatever the cell type used, major limitations of cell transplantation are the poor survival [9], [10] and the poor migration [11], [12] of transplanted cells. The massive and early death of transplanted MPCs is not fully understood. Mechanisms including anoikis, hypoxia, apoptosis/necrosis [13], [14] and immune destruction have been involved, particularly involving T cells and neutrophils [10], [15], [16] although neutrophil involvement has been challenged by more recent data [17]. Various attempts have been made to improve either survival or migration of transplanted cells, by acting on various molecular pathways including TGFβ1 [18], metalloproteases [19], urokinase–type plasminogen activator [20], heat-shock proteins [21], [22], Fibroblast Growth Factor (FGF)b, Insulin Growth Factor (IGF)-1 [23], myostatin [24], Interleukin(IL)-4 [25], Nerve Growth Factor [26], Vascular Endothelial Growth Factor [13].

Immune cells have been observed together with transplanted cells [27]. The role of neutrophils is still unclear regarding their toxicity towards MPCs [10], [17]. Macrophages (MPs) have been regularly shown to be non toxic towards MPCs; moreover, a study reported an increased MPC proliferation and differentiation in the presence of MPs [28]. Several *in vivo* and *in vitro* studies have shown that MPs are beneficial during muscle regeneration. It is known for a long time that muscle repair is associated with MP influx into the damaged area [29], [30]. Partial inhibition of monocyte/MP recruitment in muscle severely impairs muscle regeneration [31]–[34] while total inhibition of monocyte recruitment into skeletal muscle at time of injury prevents muscle regeneration [35]. Beyond MP role in phagocytosis of muscle debris, several studies have identified new trophic roles for these immune cells towards MPCs. *In vitro*, MPs stimulate MPC growth, by both the secretion of soluble factors that are mitogenic for MPCs [36]–[38] and by establishing direct anti-apoptotic cell/cell contacts [39]. Thus, MPCs establish privileged relationships with the neighbouring MPs, which in turn support their fate during regeneration. Accordingly, intramuscular administration of MP-conditioned medium improves muscle regeneration [40]. The trophic activities of MPs toward MPCs could be of interest and exploited during skeletal muscle cell transplantation. Attempting to recreate an appropriate stromal support using MPs can be useful to avoid MPC death, and improve their growth and migration. The aim of this study was to evaluate whether MPs are beneficial during the first stages of MPC transplantation (up to 5 days) into diseased skeletal muscle. We tested the hypothesis that co injection of MPCs with MPs: 1) limits the acute death of transplanted MPCs, 2) stimulates MPC growth, 3) enhances MPC migration within skeletal muscle tissue.

## Results

**Figure 1:**
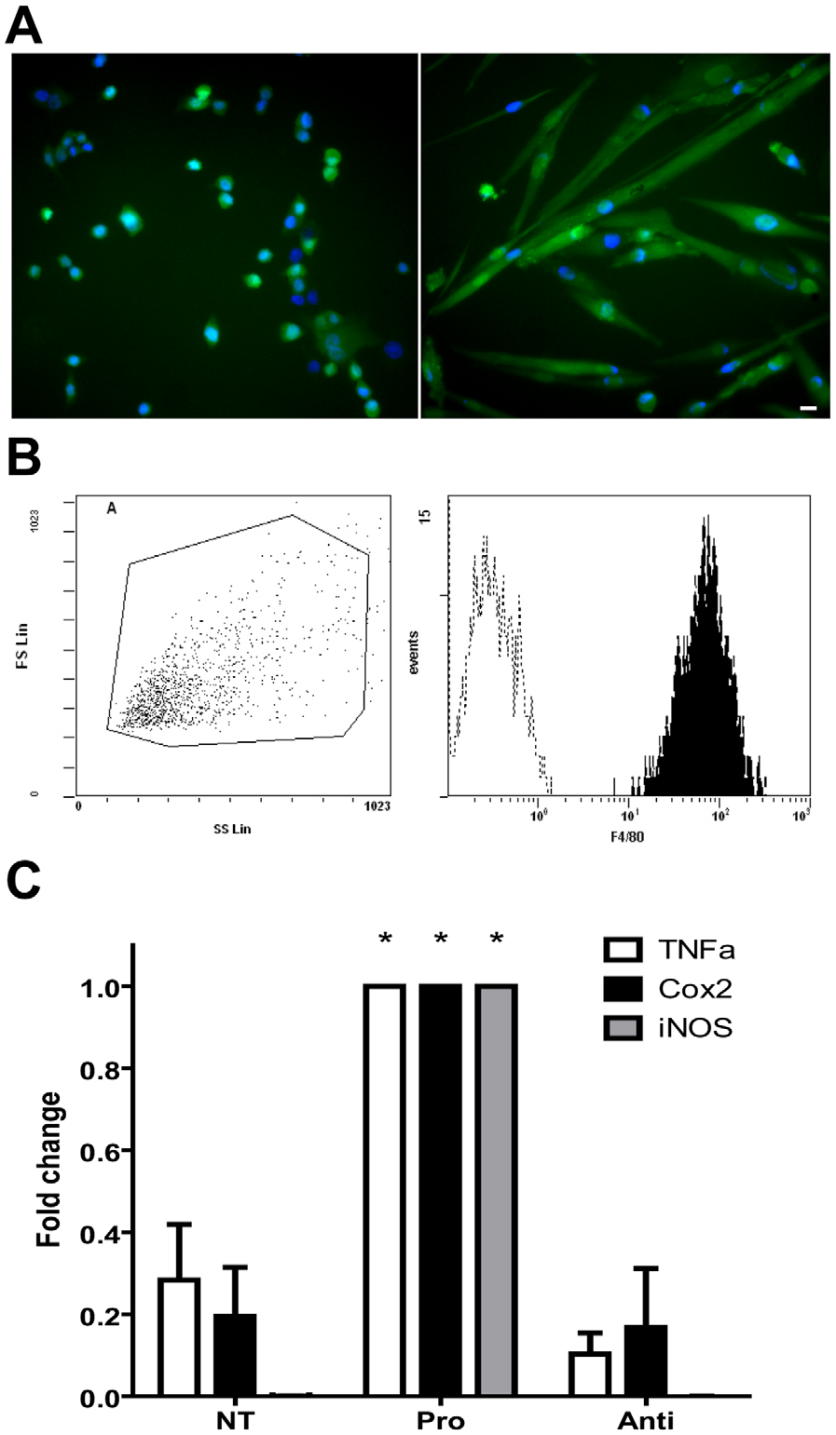
MPCs and MPs used in the study. (A) MPCs issued from Tg:CAG-GFP muscle were cultured and expanded (A, left) before their *in vitro* or *in vivo* use. These cells are capable of forming differentiated myotubes (Aright) (blue = Hoechst). Bar = 10 µm. (B) MPs were differentiated from bone marrow precursors. Differentiation into MPs is assessed by the positive labelling of more than 99% of the cells for the pan-macrophage marker F4/80. (C) Expression of iNOS, TNFα and COX2 was assessed by RT-qPCR on resting (NT) pro-inflammatory (Pro) and anti-inflammatory (Anti) MPs. Results represent mean ± sem of 4 independent RT-qPCR experiments. *P<0.05.

Murine MPCs were classically extracted from WT or from CAG-GFP mice skeletal muscle and expanded in culture (Fig. 1A) before *in vitro* or *in vivo* use. MPCs express classical myogenic markers such as Pax7 and desmin (not shown) and are capable to differentiate to form myotubes *in vitro* (Fig. 1A). MPs were differentiated from bone marrow precursors under CSF-1 stimulation. Differentiated cultures showed 99.8% of the pan-macrophage marker F4/80 positive cells (Fig. 1B), indicating that the almost totality of the cell population was composed of MPs. MPs can adopt various inflammatory profiles depending on the cues they encounter. We have previously shown that differentially activated MPs may alter MPC fate (proliferation vs. differentiation…) [35]. The purpose of the present study is to analyze the effects of MPs on the very first steps of MPC transplantation. Therefore, resting (untreated) MPs have been used in the present study. Resting MPs expressed low levels of pro-inflammatory genes such as iNOS, COX2 and TNFα, compared to pro-inflammatory MPs (Fig. 1C). We have previously shown that such resting MPs do not phagocyte MPCs but increase their proliferation and growth *in vitro* [38], [39].

### MPs Increase the Transplanted MPC Population in mdx Muscle through Decrease of Apoptosis and Increase in Proliferation

**Figure 2:**
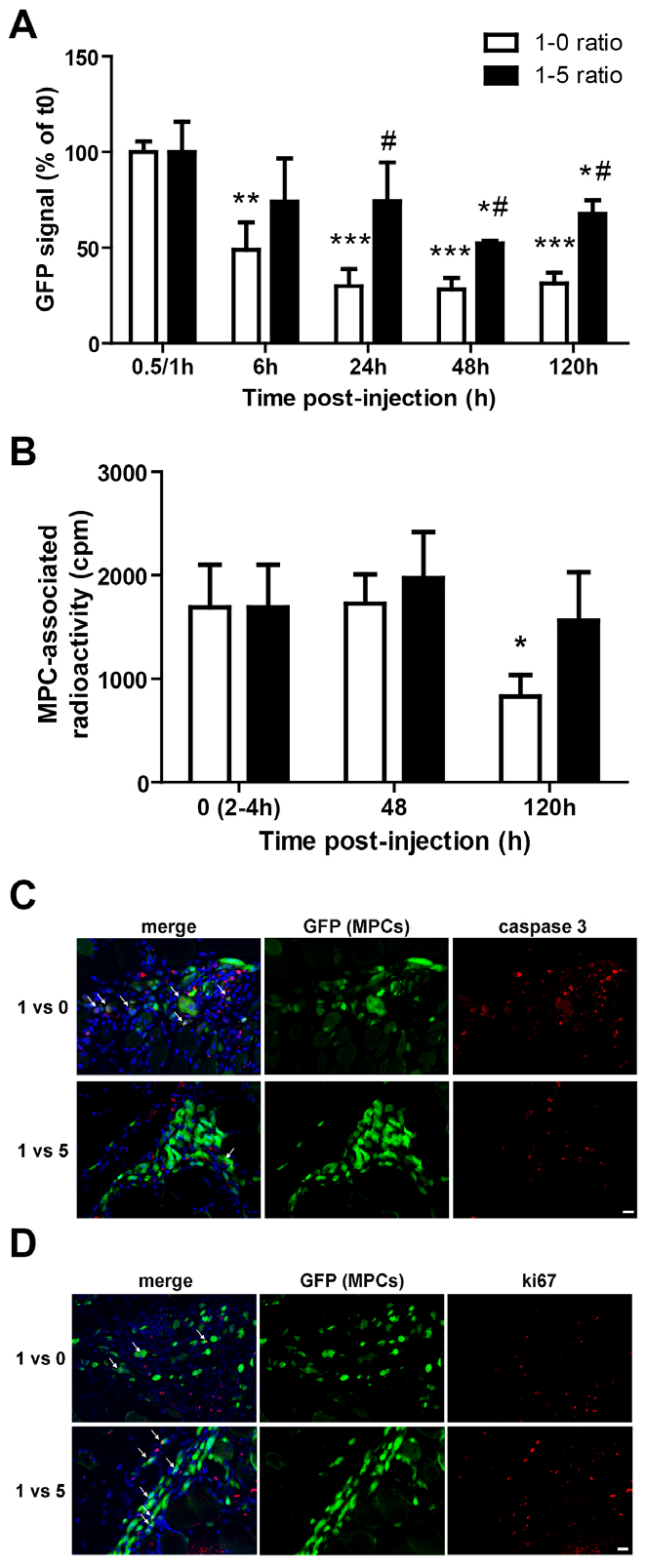
Effects of MPs on MPC engraftment in skeletal muscle. (A) GFP-MPCs were injected in mdx TA muscle with or without MPs at 1∶0 and 1∶5 ratios. At different time points after transplantation, the amount of GFP contained in injected muscles was evaluated by immunoblotting and expressed in % of the signal evaluated at time 0.5/1 h (results represent mean ± sem of 3 independent experiments). *: versus time 0.5/1 h (** = P<0.01, *** = P<0.001). #: 1∶5 versus 1∶0 ratio at each time point (# = P<0.05). (B) Radiolabelled MPCs were injected in mdx TA muscle with or without MPs at 1∶5 ratio. At 0 (2/4 h), 2 and 5 days after transplantation, radioactivity contained in injected muscles was measured and expressed in cpm (results represent mean ± sem of 4 independent experiments). *: versus d0(2/4 h) P<0.05. (C) Sections of muscles injected with GFP-MPCs with or without MPs were labelled for caspase 3. Arrows show double stained apoptotic MPCs. Bar = 10 µm. (D) Sections of muscles injected with GFP-MPCs with or without MPs were labelled for ki67. Arrows show double stained cycling MPCs. Bar = 10 µm.

We used MPCs isolated from Tg:CAG-GFP mouse in which all cells constitutively express GFP, and transplanted them with or without MPs (1 MPC for 5 MP ratio) in dystrophic mdx skeletal muscle (lower amounts of MPs were much less efficient on MPC grafting, not shown). After transplantation, GFP signal was evaluated in muscle by immunoblotting in whole muscle proteins and compared to the signal obtained in the transplanted muscle less than 1 h after transplantation. GFP signal associated with MPCs injected alone rapidly decreased during the first hours after injection (by 52% in 6 h, p<0.001), then still decreased by 42% more at 24 h. Thereafter, GFP signal remained stable until day 5 (Fig. 2A). When MPs were added, the decrease of the GFP signal was slower. GFP signal was significantly decreased compared to the 0.5/1 h time point only from 48 h (48% decrease, p<0.05) and was stabilized at day 5 (Fig. 2A). The more important effect of MPs was observed at 24 h, were the MPC-associated signal in the presence of MPs was 248% higher than without MPs. For the two last time points tested, the GFP-MPC associated signal in the presence of MPs was about 200% higher than without MPs (185 and 216% increase, respectively, p<0.05) (Fig. 2A). Endogenous GFP allows to follow all the progeny of surviving MPCs in the host muscle. The increase in GFP signal may be due to a decrease of MPC death/apoptosis and/or an increase of MPC proliferation/expansion.

To assess the first point, we evaluated survival of radiolabelled MPCs after transplantation with or without MPs into dystrophic mdx muscle. Fig. 2B shows that the radioactivity signal associated with MPCs was strongly decreased (about 50%, p<0.05) 5 days after transplantation. When adding MPs (1∶5 ratio), the amount of radioactivity recovered from the transplanted muscle at day 5 was similar to that observed at day 0 (Fig. 2B), indicating that the loss of MPCs (between 2/4 h to 5 days after transplantation) was prevented by the presence of MPs. Sections of transplanted muscles were immunolabelled for caspase 3, an effector of apoptosis. At day 5, some transplanted GFP-labelled MPCs did show caspase 3 positivity when injected alone, that is less frequently observed in the muscle cotransplanted with MPCs and MPs (Fig. 2C). These results indicate that the presence of MPs partially prevented the acute death of transplanted MPCs into skeletal muscle. To analyze whether surviving MPCs were proliferating in the transplanted muscle, immunolabelling for ki67, a cell cycle marker, was performed on muscle sections. Fig. 2D shows that in the presence of MPs, the number of MPCs positive for ki67 was increased.

We cannot exclude that a part of the GFP signal measured by immunoblotting may be associated with MPs that would have ingested some dead MPCs. However, this is unlikely to be an important phenomenon. First, if MPs would have phagocytosed large amounts of injected MPCs, the GFP signal should have decreased as a result of enzymatic digestion. Our results showed the opposite: in the presence of MPs, GFP signal was increased. Second, we never observed phagocytic features on the histological sections. Moreover colocalization of the MP marker F4/80 with GFP was not observed, indicating that transplanted GFP-labelled MPCs were not phagocytosed by MPs from day 1 to day 5 after transplantation (not shown).

### MPs and MPCs are in Close Vicinity when Cotransplanted in mdx Muscle

**Figure 3:**
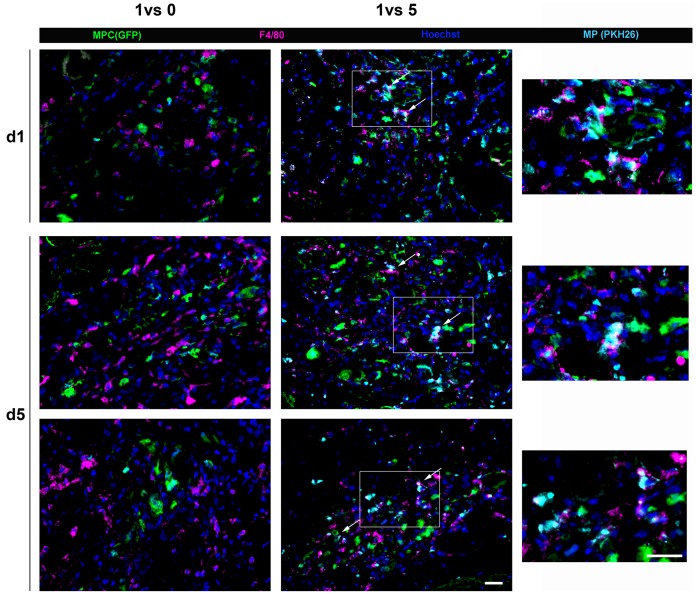
Localisation of MPCs and MPs at sites of transplantation. GFP-MPCs were injected with or without MPs which were labelled with PKH26 before the cotransplantation into mdx muscle. Immunolabellings of the pan-macrophage marker F4/80 (magenta) were performed on sections at day 1 and 5 after transplantation. Green = MPCs, cyan = PKH-labelled MPS, blue = Hoechst). Arrows show colocalization of cyan and magenta stainings (white) indicating the expression of F4/80 by transplanted MPs. Two examples from different muscles are given for day 5. Bar = 10 µm. Inserts on the right panel represent higher magnification of the fields delimited by rectangles. Bar = 20 µm.

The observation of injected muscles showed that MPCs injected alone were located near some endogenous F4/80 positive MPs, which were distributed relatively regularly within the muscle tissue, as mdx muscle is characterized by inflammation (Fig. 3). MPs were labelled with a membrane dye (PKH26) before coinjection with MPCs. Fig. 3 shows that MPCs were in very close proximity to injected MPs, all positive for F4/80, suggesting privileged interactions between the co-transplanted cells. This proximity between MPCs and MPs was observed at both day 1 and day 5 after transplantation (Fig. 3).

**Figure 4:**
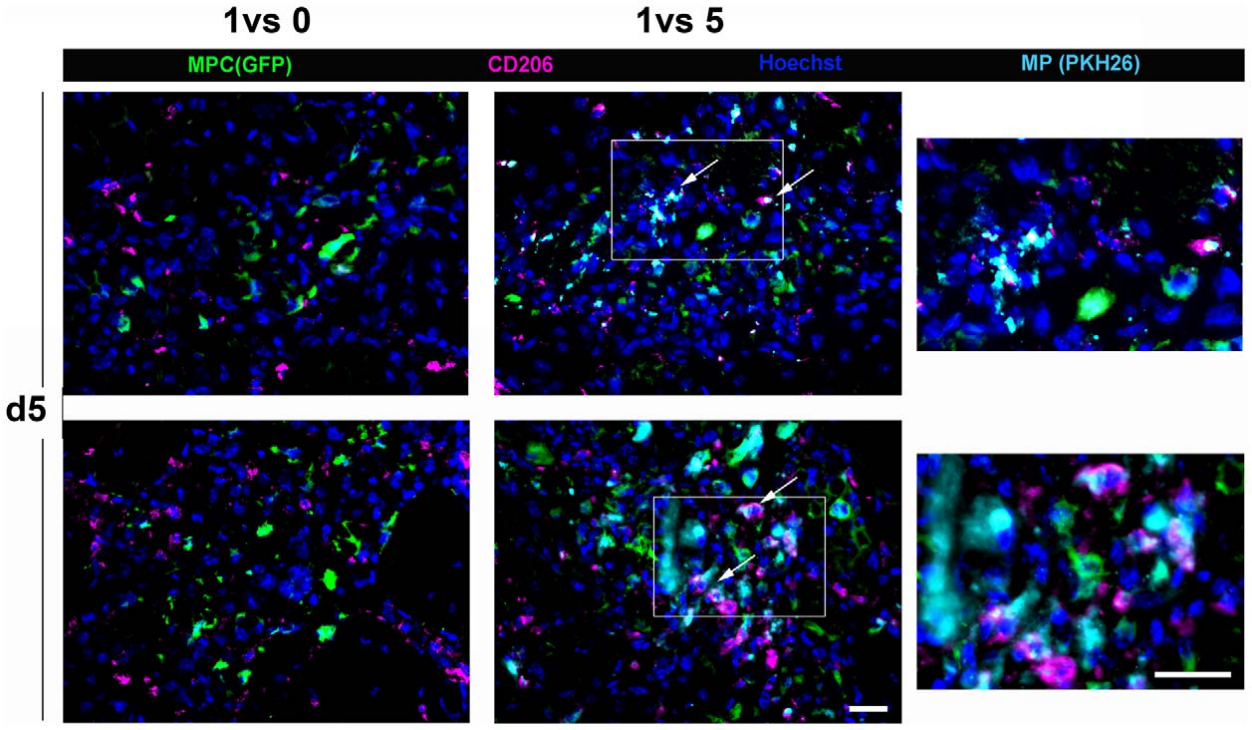
Expression of the M2 marker CD206 by MPs at site of transplantation. GFP-MPCs were injected with or without MPs which were labelled with PKH26 before the cotransplantation into mdx muscle. Immunolabellings of CD206 (magenta) were performed on sections at day 5 after transplantation. Green = MPCs, cyan = PKH-labelled MPS, blue = Hoechst). Arrows show colocalization of cyan and magenta stainings (white) indicating the expression of CD206 by transplanted MPs. Two examples from different muscles are given. Bar = 10 µm. Inserts on the right panel represent higher magnification of the fields delimited by rectangles. Bar = 20 µm.

The exact nature of MPs in mdx muscle is not yet known. Some studies suggest the presence of both pro- and anti-inflammatory MPs in mdx muscle [41], [42], although the respective amounts of these cells is not known. Transplanted muscle sections were labelled for CD206, a marker of the M2 anti-inflammatory phenotype of MPs [43]. In muscle injected with MPCs alone, part of the cells around GFP-MPCs expressed CD206 (Fig. 4), indicating that some of the endogenous MPs present a M2 phenotype. In muscle cotransplanted with GFP-MPCs and PKH-labelled MPs, we observed that the vast majority of injected MPs expressed CD206 (Fig. 4). This suggests that the close environment of co-transplanted MPCs contained higher amounts of anti-inflammatory MPs than that of MPCs injected alone.

### MPs Improve MPC Adhesion in vitro

**Figure 5:**
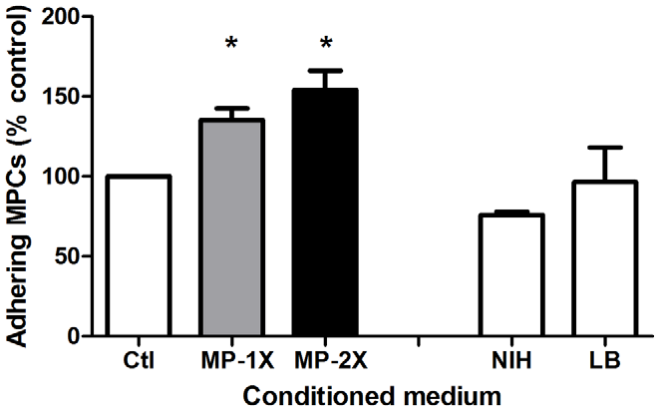
Effects of MPs on MPC adhesion *in vitro*. MPCs were seeded with or without MP-conditioned medium at various concentrations or with NIH-3T3 fibroblast-conditioned medium of B lymphocyte-conditioned medium for 5 h. Adherent cells were counted and adhesion was expressed in % of adherent cells counted in control medium (results represent mean ± sem of 3 independent experiments). * = P<0.05.

Loss of adhesion, or anoikis, may be responsible of cell death after cell transplantation [14]. To address whether MPs may have an effect on MPC adhesion, MPC were seeded in the presence of MP-conditioned medium. Fig. 5 shows that compared to control (non conditioned medium), MP-conditioned medium increased MPC adhesion in a dose dependent way (increase of 35% and 54% for medium that correspond to 1∶1.5 and 1∶3 ratio respectively, p<0.05), suggesting the delivery of trophic factors acting positively on MPC adhesion process. MP effect on MPC adhesion seemed specific since neither NIH-3T3 fibroblast- nor B lymphocyte-conditioned media induced an increase of MPC adhesion (Fig. 5).

### MPs Improve MPC Migration in vitro

**Figure 6:**
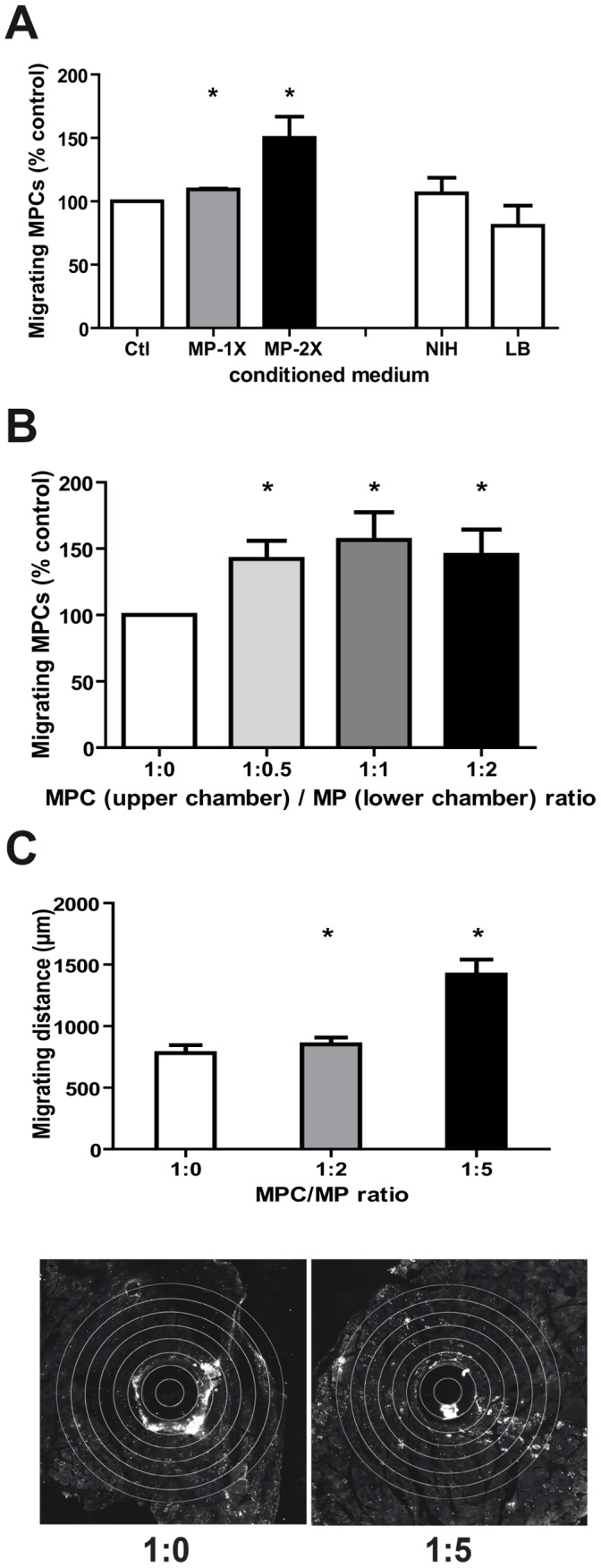
Effects of MPs on MPC migration *in vitro* and *in vivo*. (A) MPCs were seeded in the upper chamber of chemotactic inserts with or without MP-conditioned medium at various concentrations or with NIH-3T3 fibroblast-conditioned medium of B lymphocyte-conditioned medium. In (B) MPs were seeded in the lower chamber at various densities. The number of migrating cells was counted 24 h later and migration was expressed in % of migrating cells counted in control medium. (results represent mean ± sem of 3 independent experiments). * = P<0.05. (C) GFP-MPCs were injected with or without MPs at various ratios through a microtube migrating device in mdx TA muscle as described in Materials and Methods. After 48 h, migration of cells is evaluated by calculating the distance of cells from the microtube and expressed in µm. (results represent mean ± sem of 3 independent experiments). * = P<0.05. On bottom, representative micrographs show migration of MPCs from the microtube, when injected alone (1∶0) or with MPs (1∶5).

*In vitro* evaluation of the effect of MPs on MPC migration was performed using porous Boyden chamber. MPCs migrated towards MP-conditioned medium in a dose-dependent way (Fig. 6A) (increase of 10 and 50% for 1X and 2X conditions, respectively, p<0.05). Inversely, they were attracted neither by NIH-3T3 fibroblast- nor by lymphocyte B-conditioned media (Fig. 6A). When alive MPs were deposited in the lower chamber of the migrating device, migration of MPCs was also enhanced of about 50% whatever the MP density (Fig. 6B, p<0.05). This suggests that in these conditions, the continuous delivery of chemotactic factors reached the plateau from the first MP density tested. All these experiments show that MPs continuously deliver chemotactic cues for MPCs.

### MPs Improve in vivo Migration of Transplanted MPCs

*In vivo* evaluation of the effect of MPs on MPC migration was performed as previously described [23], two days after transplantation. Fig. 6C shows that MPs at a 1∶2 ratio did not significantly improve MPC migration. However coinjecting MPs at 1∶5 ratio strongly increased MPC migration by 80% (p<0.05) in mdx muscle.

### MPs Improve MPCs Engraftment in mdx Muscle

**Figure 7:**
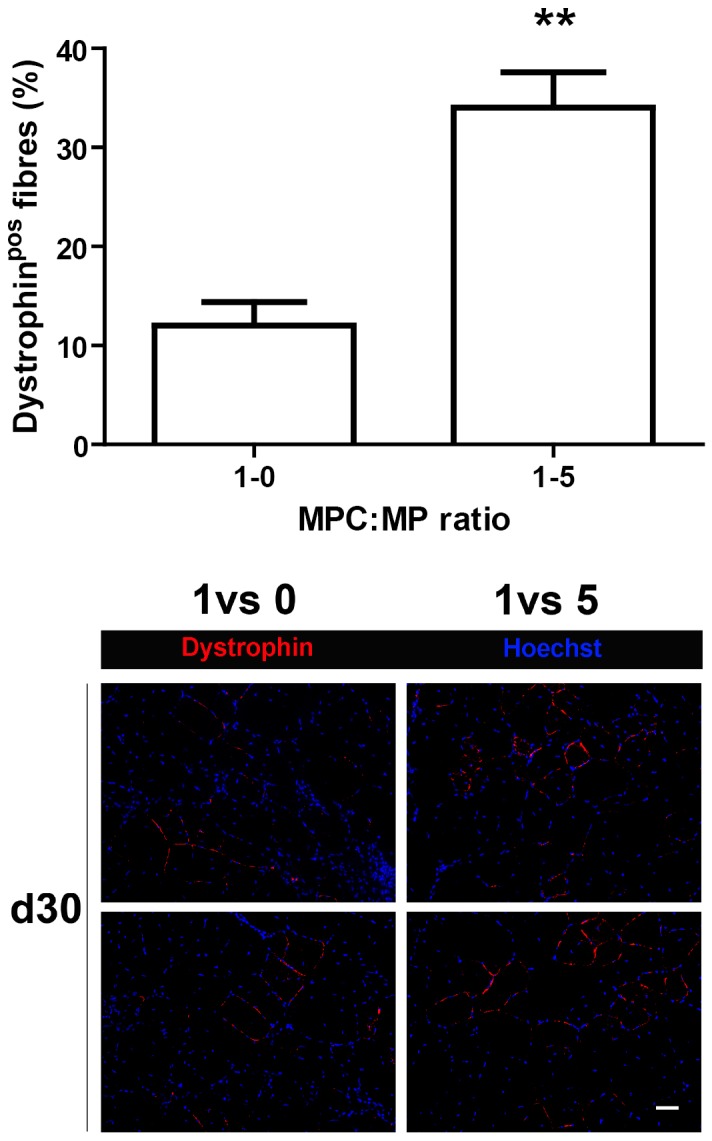
Effect of MPs on MPC participation to regenerating myofibres. GFP-MPCs were injected in mdx TA muscle with or without MPs at 1∶0 and 1∶5 ratios. 30 days after transplantation, muscle sections were analyzed for the expression of dystrophin. The number of dystrophin positive myofibres is given for one field and represents mean ± sem of 3 independent experiments). ** = P<0.01. Representative micrographs show dystrophin positive myofibres in the 1∶0 and 1∶5 conditions (two examples per condition). Bar = 10 µm.

To analyze whether the early beneficial effects of MPs on MPC transplantation have an impact on MPC contribution to regenerating myofibres, transplanted muscle were analyzed 30 days after injection of cells. Fig. 7 shows that the coinjection of MPs with MPCs enhanced the number of dystrophin positive myofibres (by 280%, p<0.01), indicating a higher participation of MPCs to muscle regeneration in the presence of MPs, likely as a consequence of the increase of the total MPC number during the few days after transplantation.

## Discussion

The present data show that MPs are beneficial during the first steps of MPC transplantation in skeletal muscle by supporting both survival, proliferation and migration of the cells.

Since the very first trials using myoblast transplantation, studies regularly show that the injected cells massively die few hours/days after injection, poorly proliferate and do not migrate within the muscle tissue. This limits the use of MPCs for therapeutic purposes to focal diseases targeting small muscles, such as Oculopharyngeal Muscular Dystrophy, Facioscapulohumeral Muscular Dystrophy or sphincter insufficiency. The reasons for the death of injected MPCs are not fully understood. Loss of cell-associated signal has been reported from the very first hours post-transplantation [44], [45] and lasts for several days [9], [10], [14]. Beside cell leakage from the muscle parenchyma soon after injection, the rapid loss of implanted cells has not been deeply investigated and is not clearly explained. Nevertheless, after several hours/day, it is likely that the cells, which are injected in huge number, are isolated from the trophic cues necessary for their survival. Indeed a recent study indicates that myoblasts better survive in host muscle when injected in lower number (10^3^ cells) than when injected at higher doses (10^5^–10^6^ cells) [46]. Thus cells are likely unable to correctly interact with their environment. Of particular interest, it has been shown that anoikis may participate to cell death after transplantation [13]. Our *in vitro* results show that MPs increase cell adhesion in a serum-free environment, suggesting that MPs may help transplanted cells to avoid anoikis. In a previous study, we have shown *in vitro* that MPs establish cell/cell interactions that rescue MPCs from apoptosis [39]. When MPCs are coinjected with MPs, they may benefit from the anti-apoptotic and pro-adhesion cues delivered by surrounding MPs. Our data are in accordance with this hypothesis since the expression of caspase-3 by transplanted MPCs is decreased in the presence of MPs. These data confirm that MPs are not deleterious for injected MPCs, as previously shown by several studies [10], [17]. Moreover, MPs likely stimulated the expansion of surviving cells. Few studies analyzed the proliferation rate of transplanted MPCs. MPC proliferation index increases by 40% when cells are co-injected with vitronectin [14]. We have previously reported a mitogenic effect of MPs on MPCs [35], [38], mainly by the delivery of mitogenic factors. Various growth factors known to stimulate MPC growth are potentially secreted by MPs including IGF-I and II, Hepatocyte Growth Factor, FGFs, Platelet-Derived Growth Factor-BB, Epidermal Growth Factor, and IL-6 [47].

Our data show that the loss of the transplanted cells (about 70% at day 5) was not totally prevented by the presence of MPs, but was partially rescued. MPs seemed to not rescue the very acute MPC loss during the first hours following injection (0–6/8 h, [44]). However, MPs partially prevented the following MPC loss (from 12–24 h, [44]). The efficiency of MPs is high, about 200%, and likely accounts for both inhibition of apoptosis and stimulation of proliferation of MPCs. Adding MPs had the highest efficiency of transplanted cell rescue when compared with other treatments, including TGFβ, heat-shock, Vascular Endothelial Growth Factor overexpression, inhibition of apoptotic signalling, that rose to 40–46% of cell survival after few days [13], [14], [18], [21].

Compared to normal regenerating muscle, it is likely that the permanent degenerating/regenerating environment of mdx muscle represents an adverse environment for the injected MPCs, including necrosis, chronic inflammation and matrix remodelling. Infiltration of immune-cells containing MPs is known in mdx muscle. Several markers expressed by MPs skewed to both pro-inflammatory and anti-inflammatory states have been shown to be expressed in mdx muscle [41], [42]. Although MP phenotypes have not been yet characterized, these studies suggest the existence of various MP subsets coexisting together within mdx muscle. These include pro-inflammatory MPs, which promote inflammatory cues in the milieu, anti-inflammatory MPs, which dampen inflammation signals and alternatively activated MPs which are associated with the fibrotic process (Chazaud, unpublished data), which respective localization in dystrophic muscle areas and functions on myogenesis are still unknown. Accordingly, we showed that only a part of endogenous MPs expressed the M2 alternative/anti-inflammatory marker CD206. By contrast, almost all transplanted MPs expressed CD206 (in accordance with their *in vitro* profile, fig. 1), and would provide a specific environment to the transplanted MPCs. Indeed, immunocolocalization showed that co-injected MPs remained in the close vicinity of MPCs, during the 5 days following injection. This proximity would favour beneficial exchanges of effectors and supporting contacts between the two cell types. This feature has been observed in another study, where stromal CD31–CD45- Side Population cells were coinjected with MPCs. SP cells increased MPC transplantation by stimulating proliferation and migration of MPCs while being located in the close vicinity of transplanted MPCs [48]. Thus, while being present in the tissue, endogenous stromal cells (SPs or MPs) are not efficient enough – not close enough? - to rapidly support cell transplantation while coinjection of exogenous cells is beneficial.

The data presented here show that MPs strongly stimulate MPC migration. *In vitro*, MPCs respond well to MP-conditioned medium or to a continuous MP gradient. This confirms the results obtained by Roberston et al [37] showing that MPCs efficiently migrate toward a MP gradient, as efficiently as toward some growth factors such as Platelet-Derived Growth Factor-BB, bFGF, TGFβ [37]. Inversely, we and others have shown *in vitro* that MPCs attract circulating monocytes and MPs [37], [38], indicating reciprocal chemotactic interactions between the two cell types. *In vivo*, the present data show that the efficiency of the MPs on MPC migration is high (+82%). This is more efficient than some treatments that have been tested including MMP2 overexpression, IL-4 treatment or T cell depletion [15], [20], [25] and as efficient as addition of IGF-1, bFGF, bFGF/fibronectin to the transplanted cells [20], [23], [49].

By performing parallel *in vitro* and *in vivo* studies, our results show that coinjection of MPs with MPCs improves their survival, expansion and migration during the few days post-transplantation. In accordance with this effects, the presence of MPs increased MPC contribution to the formation of new host myofibres (30 days after transplantation). In a different transplantation context, i.e. human MPCs coinjected in immunodeficient normal regenerating muscle, MPs have been also shown to increase the engraftment efficiency [50]. It is to note that in our experiments, the *in vitro* effects of MPs are observed at a lower MPC:MP ratio than for *in vivo* experiments. It is likely that no other cells interfere with MPs – and MPCs – and alter their function *in vitro*, and notably endogenous inflammatory cells. *In vitro*, we have shown that pro-inflammatory MPs stimulate MPC proliferation while anti-inflammatory MPs stimulate myogenesis [35]. In normal regenerating muscle, inflammation is spontaneously resolved and injected MPs accompany this process (injected pro-inflammatory MPs convert into anti-inflammatory MPs [50]). Therefore, cotransplantation of MPCs with pro-inflammatory MPs at the time of injury allows MPC to proliferate and migrate before differentiation takes place at the time of resolution of inflammation during the normal course of muscle repair [35], [50]. In the dystrophic context, there is no repair and pro-inflammatory cues are persistent and must be dampened for an improvement of muscle tissue and we provided here evidence that resting MPs provide a stromal support for MPC transplantation. In conclusion, the present work shows that MPs may be envisaged as helpers to improve the first steps of MPC transplantation into dystrophic skeletal muscle, including survival, expansion and migration in the host tissue.

## Materials and methods

### Animals

C57BL/6, Tg:CAG-GFP and mdx*4cv* mice were bred and experiments were conducted according to French and European regulations. Our animal facility is fully licensed by French authorities (number A-75-1402). The principal investigator had declared the protocols used in this study and is licensed for these experiments by the local Animal Care and Use Committees of DDPP (Direction Départementale de la Protection des Populations) (number A-75-1506). Experiments were conducted on 6–8 week old animals. Animals were deeply anesthetized with ketamin/xylazin before all surgeries.

### Myogenic Precursor Cell Culture

Murine MPCs were released from minced fragments isolated from Tibialis anterior (TA) muscle by enzymatic dissociation (Collagenase B (1%) and Dispase II (2.4 U/ml)) and cultured at low density [51] in standard conditions in DMEM/HAM F12 advanced medium (Gibco Life Technologies) containing 20% foetal bovine serum (FBS) and 10 ng/ml bFGF. Myoblast colonies were trypsinated and cultured in the same conditions until a pure population of myogenic cells (over 90% desmin + cells, staining with anti-Desmin ab8592 (1/50), Abcam) was obtained. In some experiments, MPCs were radiolabelled with 0.25 µCi/ml [methyl-^14^C] thymidin (50 mCi/mmol) in growth medium for 24 h as previously described [21].

### Macrophage Cell Culture

MPs were obtained from C57/Bl6 mouse bone marrow precursor cells as previously described [52]. Briefly, total bone marrow was obtained from mice by flushing femurs and tibiae with PBS. Cells were cultured in RPMI medium containing 10% FBS and 30% conditioned medium of L929 cell line (enriched in CSF-1, kindly provided by Carole Peyssonneaux, Institut Cochin, Paris and prepared as in [53]) during 7 days. Purity of differentiated MPs was estimated by flow cytometry after F4/80-RPE (CI:A3-1 clone, AbD Serotec) labelling. In some experiments, MPs were seeded at 30000 and 60000 cells/cm^2^ and serum-free medium was incubated for 24 h, recovered and centrifugated to obtain MP-conditioned medium at 1X and 2X concentrations. In some experiments, MPs were activated to obtain various activation states. Pro-inflammatory and anti-inflammatory states were obtained after 48 h of incubation with IFNγ (50 ng/ml) and IL-10 (10 ng/ml), respectively. In some experiments, MPs were fluorescently-labelled with the PHK26 kit (Sigma).

### Control Cell Culture

NIH-3T3 fibroblast cell line was obtained from ATCC (Vanassas, VA, USA) cultured in DMEM (high glucose) containing 10% FBS. Conditioned medium was obtained as described for MPs, at a density of 30000 cells/cm^2^. B Lymphocytes were obtained as follows: splenic cells were recovered and washed in RPMI medium containing 10% FBS and 10 µM βmercaptoethanol. B Lymphocytes were sorted by magnetic bead sorting using anti-CD45R(B220) beads (Miltenyi). Sorted B lymphocyte population (>95% pure) is used as described above to prepare conditioned medium.

### Cell Transplantation and Tissue Preparation

One million Green Fluorescent Protein (GFP) or radiolabelled-MPCs were pooled with MPs at 1∶5 ratio and homogenized in 20 µl HBSS. The cells were slowly injected in TA muscle using a glass capillary along the transversal axis in 10 to 15 trajectories as previously described [21]. At various time points after injection, muscles were removed, postfixed in 4% paraformaldehyde for 2 h, soaked in PBS and then in 30% sucrose overnight at 4°C. For histological analyses, whole muscle samples were snap-frozen in embedding medium (Tissue-Tek; Sakura, Japan) in nitrogen-chilled isopentane (–160°C) and kept at –80°C until use. 7 µm thick cryosections were treated for immunolabelling. For biochemical analyses, whole muscles were lysed to either extract proteins using Mammalian Cell Lysis Kit (Sigma) for western-blot analyses or to extract DNA and count for the radioactivity contents in a liquid scintillation counter (Mod Wallac 1409, Woodbridge, Ontario, Canada), as previously described [21].

### Western Blot

Muscle protein extract (30 µg) were subjected to SDS-PAGE then blotted on PVDF membranes and incubated with anti-GFP monoclonal antibody (1∶1000, Roche) overnight at +4°C, revealed by HRP-conjugated goat anti-mouse IgGs (1∶4000, Santa Cruz) detected by Amersham ECLplus kit (GE). Emission was recorded with a Storm Imager (GMI-Inc) and blot density was evaluated with Image J software. Ponceau staining of the membrane was used to evaluate the amount of deposited proteins (the density of 2 bands was analyzed with Image J). The intensity of GFP signal was normalized to that of Ponceau staining.

### In vivo Migration Assay

MPC migration was evaluated in mdx mouse TA muscle as previously described through a Polyethylene Microtube [23]. One million GFP-MPCs with or without MPs at different ratios (1∶0, 1∶1, 1∶2 and 1∶5), in 10 µl of HBSS were slowly injected from the proximal extremity of the polyethylene microtube with a 50-µm-tip glass micropipette. After 48 h, TA muscles were removed and entirely cut in transversal cross sections (18 µm thick). For each muscle, the cross section showing the maximum migration distance was selected to represent the migration distance from the injection site depicted by the microtube. Pictures were taken using a Nikon COOLPIX 4500 camera, and muscle cross section was reconstructed with Adobe Photoshop Software, concentric equidistant circles were surimposed on the photograph. Distance between each bold circle was 165 µm, and migration distance was measured from the external surface of the microtube up to the farthest located group of GFP cells.

### In vitro Adhesion Assay

MPCs were seeded onto gelatin-coated coverslips in the presence or absence of MP- and other cell type-conditioned media for 5 h at 37°C. Non adherent cells were removed by PBS washes, coverslips were Hoechst labelled and the number of adherent cells was counted.

### In vitro Migration Assay

MPCs were subjected to migration toward conditioned media or toward MP cultures (at 10, 20, 40000 cells/cm^2^) in 0.8 µm migration inserts (BD Falcon) for 24 h at 37°C. Non migrating cells on top of the filter were removed with a cotton swab and migrating cells (lower face of the filter) were Hoechst labelled and counted.

### qRT-PCR

Total MP RNAs were extracted using the RNeasy Mini Kit (QIAGEN). 1 µg of total RNA was reverse transcripted using Superscript II Reverse Transcriptase (Invitrogen). qPCR was carried out on LightCycler® 480 Real-Time PCR System (Roche Applied Science). Reaction mixtures had a final volume of 20 µl, consisted of 3 µl of cDNA, 10 µl of LightCycler® 480 SYBR Green I Master, and 10 µM of primers (iNOS forward CTGCTGGTGGTGACAAGCACATTT, iNOS reverse ATGTCATGAGCAAAGGCGCAGAAC, TNFα forward AATGGCCTCCCTCTCATCAGTT, TNFα reverse CGAATTTTGAGAAGATGATCTGAGTGT, COX2 forward ACTGGGCCATGGAGTGGACTTAAA, COX2 reverse AACTGCAGGTTCTCAGGGATGTGA). Amplification was performed after initial denaturation, at 95°C (10 s) 60°C (5 s) 72°C (10 s) repeated 45 times. Calculation of relative expression was determined by the LightCycler® 480 software and fold change was normalized against cyclophilin housekeeping gene.

### Immunohistolabellings

Mouse muscle cryosections were fixed with paraformaldehyde 4%, treated with triton 0.1X, saturated with FBS 20% and incubated with caspase 3, ki67, F4/80, CD206 or dystrophin antibodies (Abcam) overnight at 4°C. Primary antibodies were revealed by cy3- or cy5-labeled secondary antibodies (Jackson ImmunoResearch Laboratories, Inc.). Pictures were recorded with a DMRB Leica and a Coolsnap camera. The number of dystrophin positive fibres was counted on 8 random fields (×10 objective) per section from sections made at 3 different levels throughout the length of the muscle.

### Statistical Analyses

All experiments were performed using at least three different primary cultures or series of animals. For *in vitro* studies, the Student’s t-test was used for statistical analyses to compare each condition against the control. For *in vivo* studies, two-way ANOVA test was performed and Bonferroni post-tests were applied. P<0.05 was considered significant.
